# Interaction between Vegetarian Versus Omnivorous Diet and Unhealthy Eating Patterns (Orthorexia Nervosa, Cognitive Restraint) and Body Mass Index in Adults

**DOI:** 10.3390/nu12030646

**Published:** 2020-02-28

**Authors:** Anna Brytek-Matera

**Affiliations:** Institute of Psychology, University of Wroclaw, 50-527 Wroclaw, Poland; anna.brytek-matera@uwr.edu.pl

**Keywords:** vegetarian diet, orthorexia nervosa, cognitive restraint, body mass index

## Abstract

The objective of the present study was two-fold: Firstly, to investigate unhealthy eating patterns and body mass index among individuals following a vegetarian diet and those following an omnivorous diet. Secondly, to examine interaction between vegetarian versus omnivorous diet and unhealthy eating patterns (orthorexia nervosa, cognitive restraint) and body mass index using a structural equation modeling approach (SEM). The study included 370 participants: 188 participants following a vegetarian diet and 182 following an omnivorous diet. Unhealthy eating patterns and body mass index were measured. Our results showed that individuals following a vegetarian diet were more likely to engage in orthorexic eating behavior compared to individuals following an omnivorous diet. In addition, they had a significantly lower levels of cognitive restraint and lower body mass index than individuals following an omnivorous diet. Use of SEM method showed that: (1) following a vegetarian diet and orthorexia nervosa were directly associated, (2) following an omnivorous diet and cognitive restraint were directly related and (3) following an omnivorous diet had a greater tendency to cognitive restraint and an elevated body mass index. More research is necessary to further understand the complexity of the relationship between type of diet and unhealthy eating patterns in adults.

## 1. Introduction

In recent years, there has been an increased number of individuals following a vegetarian diet [1]. The main motives for following a free-meat diet include health, moral, economy, ecology, environment, society, culture, ethics and religion [1]. According to the American Dietetic Association, an appropriately planned vegetarian diets are healthful, nutritionally adequate, and may provide health benefits in the prevention and treatment of certain diseases [2]. For many individuals, health considerations are one of the motivations for choosing particular dietary pattern [3]. Nevertheless, in some cases, interest in healthy food consumption and health could lead to orthorexia nervosa—an obsessional focus on a diet considered to be healthy, focusing on concerns regarding the quality of food, with overly care for one’s health [4]. This unhealthy eating pattern is associated with excessive time spent preparing food, inflexible dietary rules, recurrent and persistent preoccupations related to “pure” or “clean” food, rigid avoidance of foods considered “unhealthy”, compulsive behaviors, distress at violation of food rules, as well as consequent, clinically significant, impairment (e.g., medical or psychological complications, social isolation, and/or impairment in important areas of functioning) [5,6,7]. Recently, the bidimensional nature of orthorexia nervosa was proposed: with one dimension related to healthy interest in diet and healthy behavior with regard to diet (a protective factor against emotional distress) named healthy orthorexia (HeOr) and another dimension related to a pathological preoccupation with eating healthily (a new variant of disordered eating related to negative affect) named orthorexia nervosa (OrNe) [8,9,10]. The findings have demonstrated that OrNe is more common among vegetarians and vegans, compared to people who are not adhering to a special diet [9]. In addition, the latest review of the literature [11] reveals that following a vegetarian diet was found to be associated with orthorexic eating behaviors (in 11 out of 14 studies published within the last five years). Furthermore, in seven studies (out of 14), individuals following a vegetarian diet in the general population have been found to report more orthorexic behaviors or to be at risk of developing orthorexia nervosa than those who follow an omnivorous diet [11].

For some individuals, adoption of a vegetarian diet is a socially acceptable attempt to mask their disordered eating behaviors [12]. Disordered eating is characterized by a disturbed and unhealthy eating pattern that can include restrictive dieting, compulsive eating or skipping meals [13]. Cognitive restraint is defined as the intention to constantly deliberately control food intake in order to maintain or lose weight [14]. This leads to a reduction in the intake of specific macronutrients (e.g., fats or carbohydrates) or types of foods, and not to a reduction in overall caloric intake [15]. Cognitive restraint is frequently used as a marker of pathological eating behaviors [16]. Consequences of cognitive restraint are associated with: (a) dysregulation of internal perceptions of hunger and satiety which is followed by four consecutive phases: (1) low cognitive restraint—perceived food sensations and emotions and deliberately ignorance of sensations; (2) moderate cognitive restraint—perceived food sensations and emotions and lack of following the sensations due to induced negative emotions; (3) severe cognitive restraint—not perceived food sensations and emotions and struggle with maintenance of mental control (not surrendering to the emotions induction); (4) decompensated cognitive restraint—not perceived food sensations and emotions and the eating behavior is under the control of not food-evoked emotions and induced emotions; (b) disinhibition which results in the growth in food consumption (overeating); (c) emotional dysregulation—individuals that use cognitive restraint fail to reduce their anxiety levels by eating and their eating behaviors are completely managed by not food-evoked emotions or induced by food (four consecutive phases of cognitive restraint); and (d) low self-esteem and low body satisfaction [14]. Previous studies have shown the link between vegetarianism and eating-related pathology [16]. The findings have demonstrated that vegetarian diet is associated with higher levels of cognitive restraint [17]. Some studies have presented that individuals following a vegetarian diet endorses higher levels of cognitive restraint that those following an omnivorous diet [18], whereas other studies have found no difference in cognitive restraint between these both groups [17,19,20]. Although orthorexia nervosa shares a key component with cognitive restraint (self-imposed restriction of allowed food) [10], these both should be considered as distinct constructs.

Restraint theory [21] suggests that restraint (under cognitive rather than physiological control) leads reduced sensitivity to internal cues for satiety, resulting in disinhibition and overeating in situations where cognitive control is undermined (e.g., stressful events) [22]. The cross-sectional relationship between cognitive restraint and body mass index has been examined in several studies, and positive, negative and null associations between both variables have been described [22]. Nevertheless, it is worth to pointing out that in normal weight groups increased cognitive restraint has been found to be associated with increased body mass index [23,24]. That could be explained by the fact that high cognitive restraint in normal weight individuals increases the risk of overeating tendencies when restraint is relaxed, thus leading to further increases in body mass index [22].

Previous studies have indicated that body mass index increases when a wider spectrum of animal products were consumed. The results of the European Prospective Investigation into Cancer and Nutrition (EPIC—Oxford) [25] have shown that age-adjusted mean body mass index was highest in the individuals following an omnivorous diet and lowest in the individuals following a vegan diet. In addition, individuals following a pescatarian diet, vegetarian diet and especially vegan diet had lower body mass index than those following an omnivorous diet. Other studies [26,27] have recorded similar findings. All variants of vegetarian diets (vegan, lactoovo-, pesco- and semi-vegetarian diets) were associated with lower body mass index than nonvegetarian diets. The protective effects of vegetarianism against overweight may be due to avoidance of major food groups, displacement of calories to-ward food groups that are more satiating [27].

Scholars have used a diversity of methodological approaches and different measurement tools (ORTO-15, Eating Habits Questionnaire (EHQ), Düsseldorfer Orthorexia Scale (DOS), orthorexia self-test) that had different levels of validity and reliability [11] to study orthorexia nervosa. The Orthorexia Self-Test (BOT) has not evaluated the necessary psychometric properties and the cut-off scores of a reference group. A lack the basic psychometric properties of the ORTO-15 (the most widely used self-report measure of orthorexia nervosa), a low reliability and the internal consistency has been criticized. Wherefore, taking into consideration all listed limitations, in the present study, we used the Eating Habits Questionnaire (EHQ), a new research tool developed for the measurement of orthorexia nervosa and displayed good internal consistency and test-retest reliability in a college student sample. The latest review of the literature [11] provides evidence that for having more strong evidence researchers should use the EHQ and/or the DOS instead of the ORTO-15.

**Figure 1:**
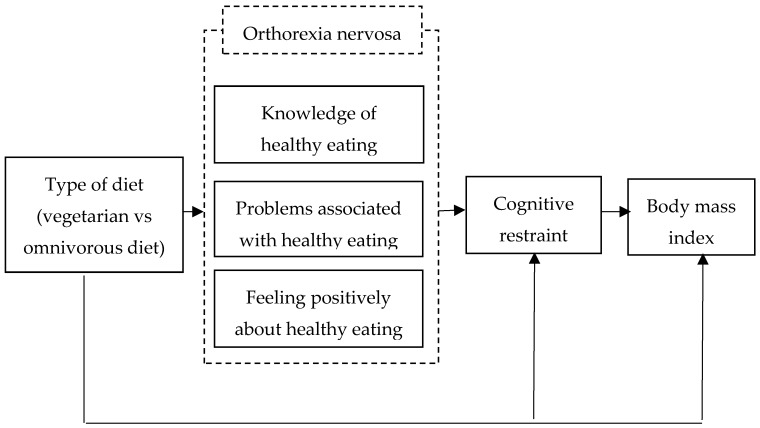
Conceptual model for the association between vegetarian versus omnivorous diet and unhealthy eating patterns (orthorexia nervosa, cognitive restraint) and body mass index in adult population.

In our latest research [28], we used a multiple linear regression to determine the predictors of orthorexia nervosa in samples with a meat-free diet. In addition, we explored the moderating role of the ethical and health reasons for following a vegetarian diet on the relation between vegan versus vegetarian diet and eating behaviors and orthorexia nervosa [28]. Therefore, the objectives of the present study were to: (1) assess unhealthy eating patterns and body mass index among individuals following a vegetarian diet and those following an omnivorous diet; (2) examine the relation between type of diet (vegetarian versus omnivorous diet) and unhealthy eating patterns (orthorexia nervosa, cognitive restraint) and body mass index using the structural equation modeling (SEM) technique. In this SEM model we postulate that: (H1) vegetarian diet and orthorexia nervosa are directly related; (H2) vegetarian diet and cognitive restraint are directly related; and (H3) body mass index is directly affected by the cognitive restraint and vegetarian diet. The conceptual model of the present study is shown in Figure 1. In the conceptual model, there are six variables, including the main dependent variable (type of diet) with the independent variable: knowledge of healthy eating, problems associated with healthy eating, feeling positively about healthy eating (three aspects of orthorexia nervosa), cognitive restraint and body mass index.

## 2. Materials and Methods

### 2.1. Participants and Study Design

We used G*Power [29] and ran a series of simulations using R statistical software to determine the sample size needed to be able conduct our least sensitive statistical tests at a power of 80% (α = 0.05, two-tailed). These analyses revealed that we would need a sample size of 191 participants (effect size = 0.3, significance level = 0.05, power = 0.95). We have assumed an attrition/unusable data rate of 20% over the study period (*N* = 268). Finally, the sample was composed of 370 participants: 188 participants following a vegetarian diet (*M*_age_ = 28.87 ± 10.32; *M*_BMI_ = 21.72 ± 3.24) and 182 participants following an omnivorous diet (*M*_age_ = 27.05 ± 8.87; *M*_BMI_ = 23.07 ± 4.98).

Data of samples were collected via online survey (SurveyMonkey). Participants were randomly selected. The notice about the research was distributed among various Silesian (Poland) institutions (vegetarian restaurants, vegan and vegetarian associations, organic grocery stores, fitness centers, dance studios, sports clubs, universities, companies) and vegetarian social networking. The announcement included a link to study information, consent procedures (anonymous and voluntary nature of participation, freedom to refuse or withdraw without penalties) and the questionnaires. Informed consent was obtained from all participants (via an online consent form). At any time and for any reason, they could refuse to answer a question or stop filling out the questionnaire and not send their data using the ‘send’ button.

Vegetarian diet was assessed through self-report. However, it is noted that individuals following a vegetarian diet were selected through predefined objective criteria (participants had to answer several questions regarding their eating behaviors and had to follow a vegetarian diet for at least 12 months). Furthermore, participants were asked to identify themselves as vegetarian (a “yes/no” item). The reason participants were excluded due to “consistency of self-defined types of diet and objective criteria” was following: discrepancy between self-description of the diet and self-identification as one of the following: vegetarian or vegan (e.g., those who described themselves as vegetarians and declared to often eat fish were eliminated). The procedure was based on the Barthel et al. [17] criteria (vegetarianism: exclusion of meat from the diet) and described in our latest publication [20].

The touch pen (worth approximately €6.00) was compensation for participation in the research. The study protocol has received the approval of a research ethics committee (no. WKEB45/03/2017). In addition, all procedures performed in this study were in accordance with the 1964 Helsinki declaration and its later amendments or comparable ethical standards. The research project was funded by the National Science Centre (NCN), Poland (Grant no. 2017/01/X/HS6/00007). The current study is part of a large project focusing on the assessment of rumination and eating behaviors in daily life among individuals with differential food preferences.

### 2.2. Outcome Measures

All participants were questioned about their age, height and weight (to calculate their body mass index) as well as their eating habits.

#### 2.2.1. The Three-Factor Eating Questionnaire (TFEQ-R18)

The TFEQ-R18 [30] assesses three different aspects of eating behaviors: cognitive restraint (conscious restriction of food intake in order to control body weight or to promote weight loss; e.g., “I consciously hold back at meals in order not to gain weight”), emotional eating (inability to resist emotional cues; e.g., “When I feel anxious, I find myself eating”) and uncontrolled eating (tendency to eat more than usual due to a loss of control over intake accompanied by subjective feelings of hunger; e.g., “When I see a real delicacy, I often get so hungry that I have to eat right away”). In the present study, we used the Polish version of the TFEQ-R18 [28] which has demonstrated satisfactory levels of internal reliability (α = 0.78 for cognitive restraint, α = 0.84 for uncontrolled eating and α = 0.86 for emotional eating). In the present study, we only used cognitive restraint scale (its Cronbach’s α values was 0.77).

#### 2.2.2. The Eating Habits Questionnaire (EHQ)

The Eating Habits Questionnaire [31] assesses cognitions (knowledge of healthy eating; “I prepare food in the most healthful way”), behaviors (problems associated with healthy eating; e.g., “I turn down social offers that involve eating unhealthy food”) and feelings (feeling positively about healthy eating; e.g., “Eating the way I do gives me a sense of satisfaction”) related to an extreme focus on healthy eating, which has been called orthorexia nervosa. The EHQ displayed good internal consistency and test-retest reliability in a college student sample [31]. The exploratory and confirmatory factor analyses support and shed further light on the construct validity of the tests. In the present study, the Cronbach’s α values of the three subscales were: 0.81 for knowledge of healthy eating, 0.82 for problems associated with healthy eating and 0.70 for feeling positively about healthy eating.

## 3. Results

### 3.1. Statistical Analysis

All analyses were carried out using the Statistical Package for Social Sciences (version 22.0 with AMOS; IBM^®^, Armonk, NY, U.S.A.). Descriptive findings for continuous data were reported using means and standard deviations which were determined using an independent sample *t*-test. Structural equation modeling (SEM) was performed to examine the structural relationship between type of diet (vegetarian versus omnivorous), unhealthy eating patterns (cognitive restraint as well as cognitions, behaviors and feelings related to orthorexia nervosa) and body mass index in adults. In the first step of the SEM, the assessment of normality (multivariate normal distribution) was performed. The asymptotically distribution-free (ADF) method was used because the critical ratio (CR) [−2, 2] and skew/kurtosis [−1, 1] for all variables did not fit in the adequate range [32]. In this work to evaluate the goodness-of-fit of a model the root mean square error of approximation (RMSEA) statistic and the comparative fit index (CFI) were used as these are the most commonly used indices [32]. The RMSEA estimates the lack of fit in a model compared to a saturated model. Values of RMSEA of 0.06 or less indicate a good-fitting model and a value larger than 0.10 is indicative of a poor model [32]. While, the comparative fit index (CFI) assesses fit relative to other models. CFI values greater than 0.90 indicate reasonably good fit of the model [32]. All statistics indicate that the goodness of fit is appropriate (Table 1).

### 3.2. Characteristics of the Study Population

The characteristics of the participants are shown in Table 2.

No significant between-group difference was observed in terms of age, (t(368) = 181; *p* > 0.05, Cohen’s d = 0.18). Whereas, there was a significant difference in body mass index between the two groups, (t(368) = − 3.09; *p* < 0.001, Cohen’s d = 0.32).

### 3.3. Comparison between Participants Following a Vegetarian Versus Omnivorous Diet: An Independent Sample t-Test

The mean (M) (and standard deviation; SD) unhealthy eating patterns and body mass index across the different diets is outlined in Table 3. There was a significant group difference in orthorexia nervosa, especially in the dimensions linked to knowledge of healthy eating, t(368) = 9.42; *p* < 0.001, Cohen’s d = 0.98, problems associated with healthy eating, t(368) = 7.48; *p* < 0.001, Cohen’s d = 0.78, and feeling positively about healthy eating, t(368) = 6.42; *p* < 0.001, Cohen’s d = 0.67. In addition, there were significant differences between the groups in cognitive restraint, t(368) = −5.30; *p* < 0.001, Cohen’s d = 0.55, and body mass index, t(368) = −3.10; *p* < 0.01, Cohen’s d = 0.32. Individuals following a vegetarian diet reported more orthorexic behaviors compared with those following an omnivorous diet. They had a significantly lower levels of cognitive restraint compared to the second group. Furthermore, individuals following a vegetarian diet had lower body mass index than individuals following an omnivorous diet.

### 3.4. Relationship between Diet, Unhealthy Eating Behaviours and Body Mass Index: a Structural Equation Modeling

**Figure 2:**
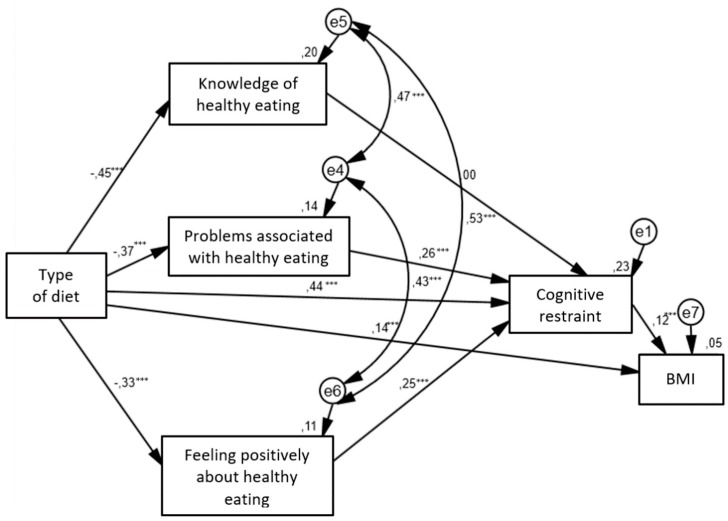
Structural equation model in adult population. 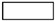
—observed variable; 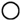
—latent variable; 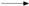
—impact of one variable on another; e—residual error in the prediction of an unobserved factor; diet—dichotomous variable (1 = vegetarian diet, 2 = omnivorous diet); BMI—body mass index; * *p* < 0.05, ** *p* < 0.01, *** *p* < 0.001. The values of standardized coefficients and squared multiple correlations (*R*^2^; over the observed variables) are presented.

The structural relationships between the different type of diets and unhealthy eating behaviors and body mass index are presented in Figure 2. For this model the estimated RMSEA is 0.03 with the 90% confidence interval (0.00; 0.097) and the *p*-value for the test of closeness of fit of 0.603. Given that the upper bound of the 90% confidence interval is less than the suggested value of 0.06 [32], and the probability value associated with this test of close fit is > 0.50, it can be concluded that the hypothesized model fits the data well. In addition, the CFI value is 0.99 which indicates an acceptable level for model fitting. The path coefficients for the path from type of diet to body mass index, from type of diet to cognitive restraint, from cognitive restraint to body mass index, from type of diet to three aspects of orthorexia nervosa, from problems associated healthy eating to cognitive restraint and from feeling positively about healthy eating to cognitive restraint were all significant. Only the path from knowledge of healthy eating to cognitive restraint was insignificant. Vegetarian diet was directly associated with higher levels of all aspects of orthorexia nervosa (knowledge of healthy eating, problems associated with healthy eating and feelings positively about healthy eating). Moreover, omnivorous diet had a greater tendency to cognitive restraint and an elevated body mass index. The indirect relationship between dietary patterns and cognitive restraint have shown that vegetarian diet contributed to stronger tendency to orthorexia nervosa. That predisposed participants to higher levels of cognitive restraint in the case of problems associated with healthy eating and feelings positively about healthy eating. Cognitive restraint was positively associated with body mass index.

Table 4 presents the coefficients with standard errors and *p*-values of the direct effects of variables on each other.

## 4. Discussion

The first objective of the present study was to evaluate unhealthy eating patterns and body mass index among individuals following a vegetarian diet and those following an omnivorous diet. Our findings confirmed that individuals following a vegetarian diet were more likely to engage in orthorexic eating behavior related to knowledge of healthy eating, problems associated with healthy eating and feelings positively about healthy eating compared to individuals following an omnivorous diet. Unlike individuals following an omnivorous diet, individuals following a vegetarian diet often turn down social events that involve eating unhealthy food, follow a diet with many rules, are distracted by thoughts of eating healthily, consider their healthy eating as a source of stress in their relationship, have difficulty finding restaurants that serve the foods they eat, place more restrictions on the foods they can eat (the EHQ’s items [31] related to problems with healthy eating). In addition, they go out less since they began eating healthily and spend more than three hours a day thinking about healthy food and following a health-food diet rigidly (the EHQ’s items [31] related to problems with healthy eating). Furthermore, they often make efforts to eat more healthily over time, feel in control when they eat healthily, feel a sense of satisfaction in eating the way they do, feel great and peaceful when they eat healthily (the EHQ’s items [31] related to feeling positively about healthy eating). Moreover, individuals following a vegetarian diet, compared to individuals following an omnivorous diet, are more informed than others about healthy eating, know more about healthy eating than other people, prepare food in the most healthful way and are convinced that their diet is more healthy than most diets, their diet is better than other people’s diets and their eating habits are superior to others (the EHQ’s items [31] related to knowledge of healthy eating). Our results are consistent with previous research using the same questionnaire (EHQ) [20,33]. Other studies (using another methods) have also indicated that individuals following a vegetarian diet reported more orthorexic behaviors than those who follow an omnivorous diet [17,34,35]. It is worth pointing out that both vegetarian diet and orthorexia nervosa share some similarities: specific food selection (consuming healthy and organic food), making eating-related issues an important area of one’s own life, focusing on quality of food intake, reduction of food intake according to specific nutrition rules, nutrition rules specifying which foods are “allowed” and which are “forbidden, rigid food rules and an inability to remain flexible in one’s eating habits” [20]. Our findings suggest that following a special diet (vegetarian diet) could prompt more focus on the quality of food and food consumption which may indicate that individuals following a vegetarian diet are more likely to display orthorexia nervosa.

Our results have shown that cognitive restraint differs between the groups with different dietary patterns. In contrast to the findings of previous studies [18,36], our results have indicated that individuals following a meat-free diet had a significantly lower control over food intake in order to influence body weight and body shape compared to individuals following an omnivorous diet. This could be explained by the fact that individuals following an omnivorous diet cognitive restraint could counteract the effects of overeating, whereas in individuals following a vegetarian diet, the overeating tendency is nearly absent. This ineffective form of weight control on food intake may results in individuals following a vegetarian diet eating less (because they are used to restrict the amount and quality of food consumed) and could lead to undereating, whereas in individuals following an omnivorous diet could lead to episodes of overeating.

Our results have shown that individuals’ following a vegetarian diet had a body mass index that was lower than the body mass index of individuals following an omnivorous diet. That could indicate that vegetarian dietary patterns may be protective in gain weight. It can be assumed that individuals following a meat-free diet engage in non-dietary lifestyle habits that promote weight loss and good health [37]. In addition, by reducing meat intake, they consume more plant-based foods that are low in saturated fat and high in fiber, both of which contribute to weight control opposite to animal products that tend to be higher in saturated fat and their intake may cause weight gain [38,39].

The second purpose of the present study was to examine the relation between type of diet (vegetarian versus omnivorous diet) and unhealthy eating patterns (orthorexia nervosa, cognitive restraint) and body mass index using the structural equation modeling (SEM) approach. To the best of our knowledge, prior research has not investigated this association. Our results indicated that vegetarian diet was directly associated with higher levels of all aspects of orthorexia nervosa (knowledge of healthy eating, problems associated with healthy eating and feelings positively about healthy eating) (H1 was confirmed). These findings are in line with prior studies in the literature that also find that higher rate of orthorexic behaviors was linked to a vegetarian diet [12,17,40,41,42]. It can be supposed that a vegetarian diet might increase the risk of developing orthorexia nervosa [11].

The direct path from vegetarian diet to cognitive restraint was not found (H2 was not confirmed). Contrary to our hypothesis, we reported that omnivorous diet and cognitive restraint were directly related. Cognitive restraint could lead to an alteration of internal perceptions of hunger and satiety and/or a disinhibition [14], therefore, it can be hypothesized that cognitive restraint in individuals following an omnivorous diet may indicate disordered eating behaviors or maladaptive eating-related attitudes.

It is worth mentioning that in the case of the indirect relationship between dietary patterns and cognitive restraint vegetarian diet predisposed to higher levels of cognitive restraint in the case of problems associated with healthy eating and feelings positively about healthy eating. One explanation of these results could be motivation for following a vegetarian diet [43]. Some individuals (especially women) with high level of cognitive restraint may adopt a vegetarian diet as a means of limiting food intake or may represent an attempt to conceal dieting behaviors form others [43]. Individuals with higher levels of cognitive restraint consume a larger amount of low-fat and calorie-reduced foods (healthy food groups), less energy, less carbohydrate and eat less food in general [44]. In addition, they may use a combination of behavioral strategies for weight control. The influential theory of dietary restraint has argued the cognitive effort required to effortfully restrict one’s intake is a causal risk factors for disordered eating behaviors [44]. This could indicate that dietary patterns that involve reduced meat intake may be employed as a socially accepted approach to engage in maladaptive weight control strategies. The adoption of a vegetarian diet after the development of an eating disorder may indicate that a vegetarian diet may play more of a role in the maintenance of eating pathology rather than being a causal factor [45]. It can be hypothesized that this mechanism could also occur in orthorexia nervosa.

Consistent with our hypothesis increased cognitive restraint was associated with higher body mass index but body mass index was not related to vegetarian diet (H3 was partially confirmed). This indicate that following an omnivorous diet had a greater tendency to cognitive restraint and an elevated body mass index.

This study has some limitations. Firstly, we conducted a cross-sectional study therefore we are unable to directly examine the causal relationship between type of diet and unhealthy eating behaviors. For investigating the causality, the future study should be focused on experimental and longitudinal studies. Secondly, we only used the subjective measures. Body mass index should be assessed by the objective methods (e.g., dual-energy x-ray absorptiometry (DXA), magnetic resonance imaging (MRI), bioelectrical impedance analysis (BIA)). Thirdly, in our study we used the structural equation modeling. It is worth mentioning that that multigroup structural equation modeling provides a powerful tool to assess the similarities and differences between different populations and equality or inequality between populations can be examined by testing whether particular parameters (e.g., factor loadings, regression coefficients between latent variables, or variances of factors and errors) in various groups are the same or different [46].

## 5. Conclusions

Our findings have demonstrated that individuals following a vegetarian diet reported more orthorexic behaviors compared with those following an omnivorous diet. Our findings have found that cognitive restraint was significantly higher in individuals following an omnivorous diet compared with those following a vegetarian diet. Furthermore, individuals following a vegetarian diet had lower body mass index than individuals following an omnivorous diet.

Use of SEM method showed that following a vegetarian diet and higher levels of all aspect of orthorexia nervosa were directly associated. More research is necessary to further understand the complexity of the relationship between type of diet and unhealthy eating patterns in adults.

## Figures and Tables

**Table 1:** Goodness of fit statistics^1^.

*χ* ^2^	*df*		*p*	*χ*^2^/*df*		Hoelter’s *N*^2^		F0^3^	RMSEA^4^		*pclose*
3.93	3		0.269	1.31		734		0.00	0.03		0.603
GFI		NFI			CFI		AIC^5^			BIC^6^	
0.99		0.99			0.99		39.93			110.37	

Note: ^1^ The current recommendations of goodness of fit (GOF) of a statistical model [32] were used in the present study: (1) *p* (for *χ*^2^) > 0.05, (2) *χ*^2^/*df* ≤ 2, (3) Hoelter’s *N* > 200, (4) F0 confidence interval includes ‘0′, (4) RMSEA (root mean square error of approximation ) < 0.06, (5) *pclose* > 0.05, (6) CFI (comparative fit index), NFI (normed fit index), GFI (goodness-of-fit index) ≥ 0.95, (7) AIC (Akaike information criterion), BIC (Bayes information criterion) hypothesized models are much closer to saturated model than independence one. ^2^ Confidence interval 95%; ^3^ F0 with approximately 90% confidence (0.00; 0.03); ^4^ RMSEA with approximately 90% confidence (0.00; 0.097); ^5^ saturated model: 42.00, independence model: 415.51; ^6^ saturated model: 124.18, independence model: 438.99.

**Table 2:** Characteristics of the study population.

Variable	Vegetarian Diet *N* = 188	Omnivorous Diet *N* = 182
	*Mean* (*SD*)	
Age	28.87 (10.32)	27.05 (8.87)
Body mass index (kg/m^2^)	21.72 (3.24)	23.07 (4.98)
	*N* (%)	
Number of meals consumed per day		
1	0 (0)	2 (1.1)
2	9 (4.8)	7 (3.8)
3	46 (24.5)	56 (30.8)
4	76 (40.4)	71 (39.0)
5	46 (24.5)	37 (20.3)
More than 5	11 (5.9)	9 (4.9)
Daily breakfast consumption		
No	7 (3.7)	10 (5.5)
Sometimes	24 (12.8)	26 (14.3)
Yes	157 (83.5)	146 (80.2)
Daily second breakfast consumption		
No	29 (15.4)	36 (19.8)
Sometimes	52 (27.7)	53 (29.1)
Yes	107 (56.9)	93 (51.1)
Daily lunch consumption		
No	4 (2.1)	5 (2.7)
Sometimes	9 (4.8)	15 (8.2)
Yes	175 (93.1)	162 (89.0)
Daily afternoon snack consumption		
No	49 (26.1)	57 (31.3)
Sometimes	76 (39.9)	63 (34.6)
Yes	64 (34.0)	62 (34.1)
Daily diner consumption		
No	6 (3.2)	7 (3.8)
Sometimes	32 (17.0)	35 (19.2)
Yes	150 (79.8)	140 (76.9)
Snacking between meals		
Never	12 (6.4)	8 (4.4)
Rarely	63 (33.5)	72 (39.6)
Sometimes	69 (36.7)	58 (31.9)
Often	29 (15.4)	22 (12.1)
Always	15 (8.0)	22 (12.1)
Between-meal snacks		
Nothing	4 (2.1)	0 (0)
Fruits	88 (46.8)	60 (33.0)
Vegetables	11 (5.9)	4 (2.2)
Sweets	39 (20.7)	75 (41.2)
Salty snacks	28 (14.9)	28 (15.4)
Other	18 (9.6)	15 (8.2)
Dietary supplement consumption		
No	69 (36.7)	121 (66.5)
Yes	119 (63.3)	61 (33.50)
Weight less method		
No	138 (73.5)	114 (62.6)
Yes	50 (26.5)	68 (37.4)
Diet	14 (7.4)	14 (7.8)
Physical activity	35 (18.6)	52 (28.6)
Laxatives	0 (0)	1 (0.5)
Vomit	0 (0)	0 (0)
Starvation diet	1 (0.5)	1 (0.5)
Daily weighing		
No	177 (94.1)	165 (90.7)
Yes	11 (5.9)	17 (9.3)
Alcohol consumption		
Never	60 (31.9)	25 (13.7)
Once a month	54 (28.7)	59 (32.4)
From twice to four times a month	53 (28.2)	68 (37.4)
From twice to three time a week	17 (9.0)	22 (12.1)
Four or more time a week	4 (2.1)	8 (4.4)
Cigarette consumption		
Never	145 (77.1)	110 (60.4)
Once a month	14 (7.4)	18 (9.9)
From twice to four times a month	5 (2.7)	17 (9.3)
From twice to three time a week	5 (2.7)	10 (5.5)
Four or more time a week	19 (10.1)	27 (14.8)
Drug consumption		
Never	171 (91.0)	157 (86.3)
Once a month	16 (8.5)	19 (10.4)
From twice to four times a month	1 (0.5)	4 (2.2)
From twice to three time a week	0 (0)	1 (0.5)
Four or more time a week	0 (0)	1 (0.5)

**Table 3:** Unhealthy eating patterns and body mass index across the dietary patterns.

Variable	Vegetarian Diet (*N* = 188)	Omnivorous Diet (*N* = 182)	*p*-Value
	*M* (*SD*)		
Knowledge of healthy eating	13.16 (2.95)	10.26 (2.98)	< 0.001
Problems associated with healthy eating	20.97 (4.41)	17.27 (5.08)	< 0.001
Feeling positively about healthy eating	11.22 (2.69)	9.46 (2.57)	< 0.001
Cognitive restraint	6.99 (3.85)	9.09 (3.75)	< 0.001
Body mass index	21.72 (3.24)	23.07 (4.97)	< 0.01

**Table 4:** Coefficient, standard error and *p*-value of the structured equation modeling (SEM) approach model.

Variable	Coefficient	Standard Error	*p*-Value
Omnivorous diet	-	-	-
Knowledge of healthy eating	−7.73	0.49	< 0.001
Problems associated with healthy eating	−9.74	0.30	< 0.001
Feeling positively about healthy eating	−6.77	0.27	< 0.001
Cognitive restraint	0.43	0.42	< 0.001
Body mass index	0.14	0.40	0.004
Knowledge of healthy eating			
Cognitive restraint	0.5	0.7	> 0.05
Problems associated with healthy eating			
Cognitive restraint	4.57	0.4	< 0.001
Feeling positively about healthy eating			
Cognitive restraint	4.33	0.8	< 0.001
Cognitive restraint			
Body mass index	2.33	0.5	0.02
